# Statistical and electrical properties of the conduction electrons of a metal nanosphere in the region of metal-insulator transition

**DOI:** 10.1186/1556-276X-9-174

**Published:** 2014-04-10

**Authors:** Vitaly V Datsyuk, Iryna V Ivanytska

**Affiliations:** 1 Department of Physics, Taras Shevchenko National University of Kyiv, 64, Volodymyrska Str., Kyiv 01601, Ukraine

**Keywords:** Nanoparticle, Metal-insulator transition, Conductivity, Capacitance

## Abstract

Statistical and electrical properties of the conduction electrons of a silver or gold sphere with a radius from 1 to 2 nm are shown to differ drastically from the properties of electrons in a bulk metal sample. If the radius of a noble metal sphere decreases from 10 to 1 nm, its conductivity oscillates around the bulk metal value with increasing amplitude and drops at the 'magic’ numbers of electrons. These numbers are equal to 186, 198, 254, 338, 440, 556, 676, 832, 912, 1,284, 1,502, and 1,760, in agreement with various experimental data. We show that the conductivity and capacitance of a metal nanosphere can be changed by several orders of magnitude by adding or removing just a few electrons.

## Background

In the past, a measurement of optical absorption by silver nanoparticles embedded in glass showed that the particles had normal metallic properties when their diameters were decreased down to 2.2 nm [1]. Contrary to this finding, metal particles with sizes below 2 nm cannot be conducting according to more recent papers [2,3]. Very recently, it was understood that the metal-insulator transition (MIT) is gradual so that particles with certain 'magic’ numbers of electrons become insulating while others remain conducting [4]. If electrons move inside a sphere, then the numbers 186, 198, 254, 338, 440, 556, 676, 832, 912, 1,284, 1,502, and 1,760 are known to be 'magic’. It was experimentally found that the above numbers are indeed magic for clusters of many metals [5-16]. This allows one to consider the motion of electrons in a spherical jellium [8,12,17,18].

We recently studied statistical properties of 500 to 2,000 free electrons confined in a spherical potential well with a radius from 1.2 to 2 nm. The averaged occupation numbers of the electron energy levels and the variances of the occupation numbers were computed for both isolated metal nanoparticles and those in equilibrium with an electron bath. The sum of the variances of all occupation numbers was found to depend on the number of electrons nonmonotonically dropping by a few orders of magnitude at 'magic numbers’ of electrons. Here, we show how the statistical properties of the conduction electrons are related with the electrical properties of metal nanoparticles. Calculations of the DC conductivity and capacitance of single nanometer-sized noble metal spheres are reported. We predict a transistor-like behavior of a single nanoparticle when an additional charge of the particle drastically changes its conductivity and capacitance.

## Methods

### Statistical and transport models

The electron statistics and capacitance of metal nanoparticles are investigated by the Gibbs ensemble method. The transport properties of electrons are studied using a quantum-mechanic generalization of a formula that can be obtained by solving the Boltzmann transport equation.

The Boltzmann transport equation (BTE) is widely used in modern physical kinetics [19,20] even at nanoscales [21,22]. In our previous work, we solved BTE and determined the response of the conduction electrons on an electromagnetic wave allowing for dependence of the distribution function on the wavenumber. Then, we obtained the spatially dispersive permittivity of metal and applied a generalized Mie theory [23] to calculate spectra of light extinction by silver nanospheres. Because of excitation of the rotationless (longitudinal) plasmon-polariton waves, the frequency of the Fröhlich resonance *ω*=3.5 eV [24] was found to increase with decreasing the radius *a*, so *ω*=3.635 eV for particles with *a*=1.5 nm and *ω*=3.73 eV at *a*=1 nm (see Figure two of [25]; a similar dependence of *ω* on *a* as well as the formulas of [23] is found in a recent paper [26]). The blueshift by 0.15 eV and the width of the plasmon resonance calculated for a particle beam created by Hilger, Tenfelde, and Kreibig [27] were in excellent agreement with the experimental data. We concluded that silver clusters with *a*<1 nm do not contribute into the extinction spectra of the beams. However, it was not possible to establish whether the contribution of these ultrathin particles into the integral extinction spectrum vanished due to MIT.

### Energies of the conduction electrons

It is a common practice to assume that the conduction electron energy *ε* in metal is a function of the continuous quasi-momentum p=ℏk. This approach can be used to model the properties of bulk metals in which an electron state *s* can be described by a set of four quantum numbers, $s=s_{z},\;p,$ where $s_{z}$ is a projection of the electron spin. However, if an electron moves inside a microscopic sphere, the vector **p** can no longer describe an electron state. The set *p*_*x*_,*p*_*y*_, and *p*_*z*_ has to be replaced by a set of the radial *q*, angular momentum *l*, and magnetic *m* quantum numbers. Then, integrals over **p** should be replaced by sums over the electron states *s*, according to the following rule: $\underset{s}{\sum }=\frac{2V}{h^{3}}\int dp$, where *V* is the volume of the body and *h* is the Plank constant. In this study, we used discrete sets of the electron energies *ε*_*s*_.

### Shape of metal nanoparticles

**Figure 1:**
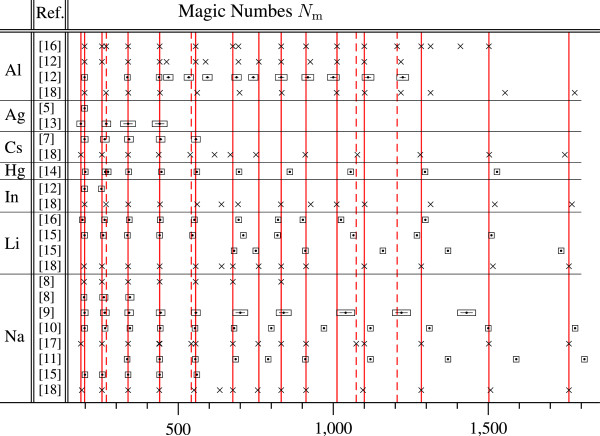
**Experimental (centered boxes with error bars) and theoretical (crosses) 'magic’ numbers of electrons in metal clusters.** Solid grid lines indicate *N*_m_= 186, 198, 254, 338, 440, 556, 676, 760, 832, 912, 1,012, 1,100, 1,284, 1,502, and 1,760. Dashed grid lines indicate *N*_m_= 268, 542, 1,074, and 1,206.

Every set of the electron energies *ε*_*s*_ was obtained at a certain number *N* of electrons confined in a potential well of an ideal spherical shape. We used the parameters of bulk silver and gold [28], namely, the depth of the potential well *U*=9.8 eV and the Wigner-Seitz radius *r*_**s**_=0.16 nm. Because of the spherical symmetry of the electronic wave functions, different physical processes have peculiar features at magic numbers of electrons. The magic numbers, which we determined for perfect noble metal spheres, are presented in the 'Background’ Section of this paper. Surprisingly, almost the same values were determined in various experiments working with particles of different metals, even though no special efforts were made to produce perfect spheres. In addition, different theoretical papers also reported similar magic numbers, according to Figure 1. This means that effects associated with the peculiarities of the spacing of *ε*_*s*_ in spherical nanoparticles are sensitive neither to surface distortions nor the values of the parameters *U* and *r*_s_.

## Results and discussion

### Variances of the occupation numbers

**Figure 2:**
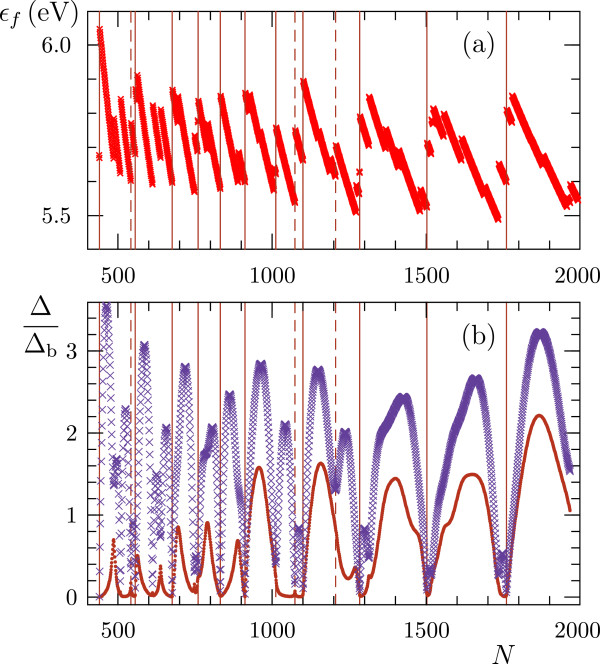
**Fermi energies and variances of the occupation numbers of electronic states of single Ag or Au spheres. (a)** Fermi energy as a function of the number *N* of conduction electrons. **(b)** Sums of the variances *Δ* normalized to the bulk metal value *Δ*_b_ in canonical ensembles (points) and grand canonical ones (crosses). The grid lines are the same as in Figure 1.

In our previous work [29], we reported statistical properties of the conduction electrons in isolated metal nanospheres. To study the systems with a fixed number of electrons, the method of the canonical ensemble was applied. The averaged occupation numbers 〈*n*_*s*_〉, variances of the occupation numbers $\langle n_{s}^{2}\rangle -{\langle n_{s}\rangle }^{2}$, and sums of the variances $\Delta =\underset{s}{\sum }\left(\langle n_{s}^{2}\rangle -{\langle n_{s}\rangle }^{2}\right)$ were computed and discussed. In [29], we also examined the properties of the conduction electrons in grand canonical ensembles where the chemical potential *μ*_0_ was fixed. Figure 2 represents the values of *Δ* calculated at fixed *N* (canonical ensembles) and *μ*_0_ (grand canonical ensembles). The sum of the variances depends on the number of electrons nonmonotonically dropping by several orders of magnitude at magic numbers of electrons. The decrease in *Δ* can occur if (i) the distance between the Fermi level and the neighboring higher energy level, *ε*_*f*+1_-*ε*_*f*_, is large compared to the thermal energy and (ii) the Fermi level is fully occupied at absolute zero temperature. Addition of one atom to a particle with *N*_m_ conduction electrons results in a substantial increase in the Fermi energy, as is evident from Figure 2a. If a particle has a magic number of electrons, the chemical potential lies in the gap between the distant energy levels, so the number of the current carriers is greatly reduced. The influence of this effect on the electrical properties of the metal nanoparticles is studied below.

### Conductivity

The response of the conduction electrons of metals to an infrared and far infrared radiation is well described by a Drude dielectric function [30]. In the corresponding limit of small emission wavenumbers, this function can be derived by using either a quantum theory by Lindhard [31] or the classical Boltzmann transport equation [32] (see derivations in [20]). The permittivity of metal is expressed through the conductivity *σ* that, in the DC case, can be calculated from the following equation:

(1) $$\sigma =-\frac{2}{3}\;\frac{e^{2}}{m_{e}}\;n_{e}\;\frac{\int \varepsilon^{\frac{3}{2^{}}}\;\frac{\mathrm{\partial f}}{\partial \varepsilon }\;\tau \left(\varepsilon \right)\;d\varepsilon }{\int \varepsilon^{\frac{1}{2^{}}}\;f\left(\varepsilon \right)\;d\varepsilon },$$

where *e* and *m*_*e*_ are the charge and effective mass of the electron, respectively, *n*_*e*_=*N*/*V*, *ε*=*p*^2^ / (2 *m*_*e*_),*f*(*ε*) is an equilibrium electron energy distribution function, and *τ* is the relaxation time. From Equation 1, the classical result $\sigma_{b}=\frac{e^{2}}{m_{e}}\;n_{e}\;\tau$ is obtained at *τ*=const and any *f*(*ε*) finite at *ε*=0 and vanishing at *ε*→*∞*. The formula for *σ*_b_ can also be derived by substituting a zero-temperature Fermi-Dirac distribution function into Equation 1.

A generalization of Equation 1 for discrete energy levels gives the following formula:

(2) $$\sigma =\frac{2}{3}\;\frac{e^{2}}{m_{e}}\;\frac{1}{V}\;\underset{s}{\sum }\frac{\varepsilon_{s}}{\text{kT}}\;\left[1-\langle n_{s}\rangle \right]\;\langle n_{s}\rangle \;\tau \left(\varepsilon_{s}\right),$$

where 〈*n*_*s*_〉 is the averaged occupation number of the state *s*. We tested Equation 2 by computing the normalized conductivity defined at constant *τ*,

(3) $$S\left(N\right)=\frac{\sigma }{\sigma_{b}}=\frac{2}{3\;N}\;\underset{s}{\sum }\frac{\varepsilon_{s}}{\text{kT}}\;\left[1-\langle n_{s}\rangle \right]\;\langle n_{s}\rangle .$$

**Figure 3:**
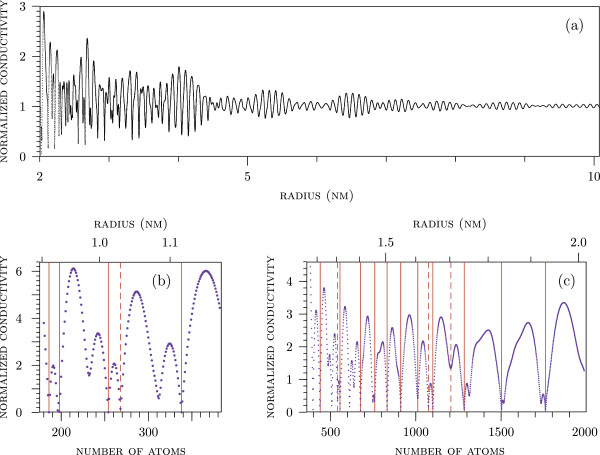
**Normalized DC conductivity.(a)** Normalized DC conductivity vs rigid-wall sphere radius *a*=*r*_s_*N*^1/3^ at *N* from 2,000 to 2.5×10^5^. Normalized DC conductivity of a neutral silver or gold sphere at **(b)***N*= 180 to 382 and **(c)***N*= 382 to 2,000. The grid lines are the same as in Figure 1.

The equality S=1 should hold for 'large’ particles since properties of a macroscopic body are independent of the boundary conditions for the electron wave function. The calculations were performed by using sets of *ε*_*s*_ for *N* free electrons confined in a spherical potential well with the radius *a*=*r*_s_*N*^1/3^, where *r*_s_=0.16 nm. Figure 3a presents the results obtained at *N* in the range from 2,000 to 2.5×10^5^,*T*=300 K. There are pulsations of S vanishing as sphere radii increase above 9 nm that corresponds to *N*>2×10^5^. Therefore, Equation 2 works well, and particles with *a*≥10 nm can be regarded as macroscopic. The left-hand side of the curve in Figure 3a (at *a* from 2 to 4.5 nm, i.e., *N* from 2,000 to 20,000) shows the oscillations of S(a) with the amplitude increasing with the decrease of *a*.

The conductivity S at *N*= 200 to 2,000 was calculated by using more realistic values of *ε*_*s*_ found for a spherical potential well with the parameters of silver and gold. According to Figure 3b,c, the value of S is not a monotonic function of *N* and drops sharply when *N* is equal to one of the magic numbers *N*_m_. The appearance of magic numbers is a general property of fermionic systems. In this paper, the magic numbers of the conduction electrons are identified by the dips in the conductivity S(N). The values of *N*_m_ and $S\left(N_{m}\right)\times 100$ are listed in Table 1. The found values of *N*_m_ are in excellent agreement with the experimental and theoretical magic numbers of clusters of many metals according to Figure 1.

**Table 1:** Normalized conductivity (%) calculated for an Ag or Au particle with a magic number of atoms

			***N***_***m***_				
	**186**	**198**	**254**	**338**	**440**	**676**	**912**
S (%)	0.03	2.6	0.01	0.005	0.37	5.6	4.1

All the experimental numbers *N*_m_ in Figure 1 were obtained by using the mass spectroscopy from dips in the mass spectra. For example, Katakuse and co-workers [6] found magic numbers of atoms equal to 197 for negative cluster ions of silver (Ag)*n*- and 199 for positive ${\left(\text{Ag}\right)}_{n}^{+}$ cluster ions. Other magic numbers of atoms were 137 for ${\left(\text{Ag}\right)}_{n}^{-}$, ${\left(\text{Au}\right)}_{n}^{-}$, and ${\left(\text{Cu}\right)}_{n}^{-}$ and 139 for ${\left(\text{Ag}\right)}_{n}^{+}$, ${\left(\text{Au}\right)}_{n}^{+}$, and ${\left(\text{Cu}\right)}_{n}^{+}$. In these cases, the negatively and positively charged and neutral particles had the same magic numbers of electrons, *N*_m_= 198 and 138. Thus, the anomalous properties of the metal nanoparticles in the experiments [5-15] are determined by electron motion [29] but not their atomic structure. Moreover, the model of single electrons trapped in a spherical potential well was shown to be adequate [6] though the shape of the clusters obtained by the bombardment of metal sheets with Xe ions was not controlled.

A nonlinear dependence of S on *N* can occur even in a single sphere if *N* varies around *N*_m_. To examine electric properties of a single charged nanoparticle, let us consider a sphere in thermal equilibrium with a reservoir of electrons, so the electrochemical potential *μ*=*μ*_0_+*eϕ* is constant inside the sphere; here *μ*_0_ is the chemical potential of the neutral sphere and *ϕ* is the electric potential. For a fixed *μ*, we determined $N=\underset{s}{\sum }\langle n_{s}\rangle$ by using the Fermi-Dirac occupation numbers and computed the charge of the sphere *Q*=*e* (*N*-*N*_0_), where *N*_0_ is the number of electrons in the neutral particle. We calculated the quantities *Q* and S for a charged 336-atom Ag or Au nanoparticle. We found that the 336-atom particle holds two extra electrons when the value *ϕ* changes in a wide range of about 0.6 V. If the mean number of electrons in the particle is equal to 338, then $S=4.4\times 10^{-5}$. The normalized conductivity of the neutral sphere is found to be S=0.78; $S(338)/S(336)=5.7\times 10^{-5}.$ In the considered example, the neutral sphere is conductive, but the charged one with two extra electrons turns out to be an insulator.

### Capacitance

A parameter that describes the dependence of *Q* on *ϕ* is the electric capacitance

(4) $$C(N)=\frac{dQ}{d\phi }.$$

A straightforward calculation of the derivative of *Q* gives the capacitance of the charged particle with 338 electrons *C*=6.1×10^-22^ F that is much lower than *C*=1.1×10^-17^ F of the neutral 336-atom sphere. The change in the capacitance *C*(338)/*C*(336)=5.3×10^-5^ is similar to the the correspondent change in the conductivity.

By calculating the derivative of *Q* in Equation 4 at *N* defined through the Fermi-Dirac occupation numbers, we get

(5) $$C=e^{2}\;\frac{\Delta }{\text{kT}},$$

where *Δ* is the sum of the variances of the occupation numbers shown in Figure 2 by crosses. Equation 5 expresses the relation between the reaction of the conduction electrons to the electric field and the fluctuations of the occupation numbers of the electron states. Thus, the peculiarities of spacing and degeneracy of the electronic energy levels have similar effects on the statistical and electrical properties of a nanometer-sized particle.

During the calculations we neglected Coulomb effects. These effects are as follows. When an electron leaves a neutral metal sphere, it overcomes the attraction of the positive charge remaining on the sphere. Consequently, the work function increases by the value *ΔU* = 0.54/*a*(nm) eV [33]. For example, *ΔU* ≃ 0.5 eV for a 338-atom noble-metal sphere. In addition, Coulomb forces should change the binding energy of the electron in a charged sphere compared to that in the neutral one. Though the change in *U* can be large, it should not be critical to the effects studied in this paper. Indeed, they depend on the value of *ε*_*f*+1_-*ε*_*f*_, but this difference is a weak function of *U*. For example, for a noble metal sphere with 338 conduction electrons, we get *ε*_*f*+1_-*ε*_*f*_=0.69 eV at *U*=9.8 eV, and *ε*_*f*+1_-*ε*_*f*_=0.74 eV if *U*→*∞*.

## Conclusion

In conclusion, the statistical properties, conductivity, and capacitance of a single nanometer-sized metal sphere depends very strongly on the number of conduction electrons *N* in the range from 200 to 2,000. In particular, the DC conductivity drops by several orders of magnitude if *N* is equal to one of the magic numbers. For instance, addition of two electrons to a 336-atom noble metal sphere should reduce both the conductivity and capacitance of the particle by four orders of magnitude.

## Competing interests

The authors declare that they have no competing interests.

## Authors’ contributions

VVD and IVI together carried computations, analyzed results, and prepared the manuscript. Both authors read and approved the final manuscript.
